# Neuropsychiatric symptoms and seizure related with serum cytokine in epilepsy patients

**DOI:** 10.1038/s41598-022-10865-x

**Published:** 2022-05-03

**Authors:** Hye-Rim Shin, Kon Chu, Woo-Jin Lee, Han Sang Lee, Eun Young Kim, Hyoshin Son, Jangsup Moon, Narae Kim, Ki-Young Jung, Keun-Hwa Jung, Soon-Tae Lee, Kyung-Il Park, Sang Kun Lee

**Affiliations:** 1 Department of Neurology, Dankook University Hospital, Cheonan, Chungnam Republic of Korea; 2 Department of Neurology, Seoul National University College of Medicine, Seoul, Republic of Korea; 3 Department of Neurology, Seoul National University Hospital, 101 Daehak-ro, Jongno-gu, Seoul, Republic of Korea; 4 Department of Neurology, Seoul National University Bundang Hospital, Seongnam-si, Gyeonggi-do Republic of Korea; 5 Center for Hospital Medicine, Seoul National University Hospital, Seoul, Republic of Korea; 6 Department of Neurology, Sejong Chungnam National University Hospital, Sejong, Republic of Korea; 7 Department of Genomic Medicine, Seoul National University Hospital, Seoul, Republic of Korea; 8 Department of Neurology, Seoul National University Hospital Healthcare System Gangnam Center, 152, Teheran-ro, Gangnam-gu, Seoul, Republic of Korea

**Keywords:** Neuroimmunology, Epilepsy

## Abstract

Neuroinflammation contributes to epileptogenesis and ictogenesis. Various signals of neuroinflammation lead to neuronal hyper-excitability. Since an interplay between epilepsy, psychiatric comorbidities and neuroinflammation has been suggested, we explored psychiatric symptoms in epilepsy patients, and the relationship with neuroinflammation. We screened epilepsy patients who were admitted for video-EEG monitoring between July 2019 and December 2020. Enrolled patients were asked to respond to neuropsychiatric questionnaires (Hospital Anxiety and Depression Scale (HADS) and Neuropsychiatric Inventory-Questionnaire (NPI-Q)) on admission. Serum cytokines (IL-1β, IL-2, IL-6, IFN-γ, CCL2, and CCL5) were measured by ELISA on admission, and within 6 h after a seizure. We enrolled 134 patients, and 32 patients (23.9%) had seizures during monitoring. Cytokine levels did not change after seizures, but IL-2 and IL-6 increased in cases of generalized tonic–clonic seizures. The HADS-A score was lower in Q4 of CCL5 (*p*-value = 0.016) and anxiety was also less common in Q4 of CCL5 (*p*-value = 0.042). NPI-Q question 4 (depression) severity was higher in CCL2 (*p*-value = 0.024). This suggested that psychiatric symptoms may also be related to inflammatory processes in epilepsy patients. Further large, standardized studies are necessary to underpin the inflammatory mechanisms in epilepsy and psychiatric symptoms.

## Introduction

Epilepsy is a common neurologic disorder which presents with recurrent unprovoked epileptic seizures^1^. Various types of brain injury cause epilepsy, including traumatic brain injury, hypoxic encephalopathy, and stroke^2^. Many previous studies have suggested that inflammatory processes contribute to epileptogenesis^3–6^. After nervous system damage, cyclooxygenase activity and proinflammatory cytokines are also activated, leading to a hyper-excitatory condition in the brain^3–8^.

Psychiatric disorders are more common in epilepsy patients than in the general population^9–11^, and about 20–30% of epilepsy patients have depressive and anxiety disorders^11^. Depression is a common comorbidity in epilepsy patients, and the relationship between the two disorders suggests that epilepsy and depression may share a common mechanism^12–15^. There is growing evidence that inflammation may also play an important role in the pathogenesis of psychiatric disorders, including mood disorders, anxiety disorders, and schizophrenia^16–18^. However, there are few clinical studies demonstrating relationships between inflammation with seizures and psychiatric symptoms in epilepsy patients^12,13^.

In this study, we aimed to determine whether neuroinflammation is related to seizure and comorbid psychiatric disorders in epilepsy patients. To demonstrate this hypothesis, we measured serum cytokine in epilepsy patients and analyzed the correlation between seizures and psychiatric symptoms. We also evaluated the correlations between serum cytokines and psychiatric symptoms.

## Results

### Patient characteristics

**Figure 1:**
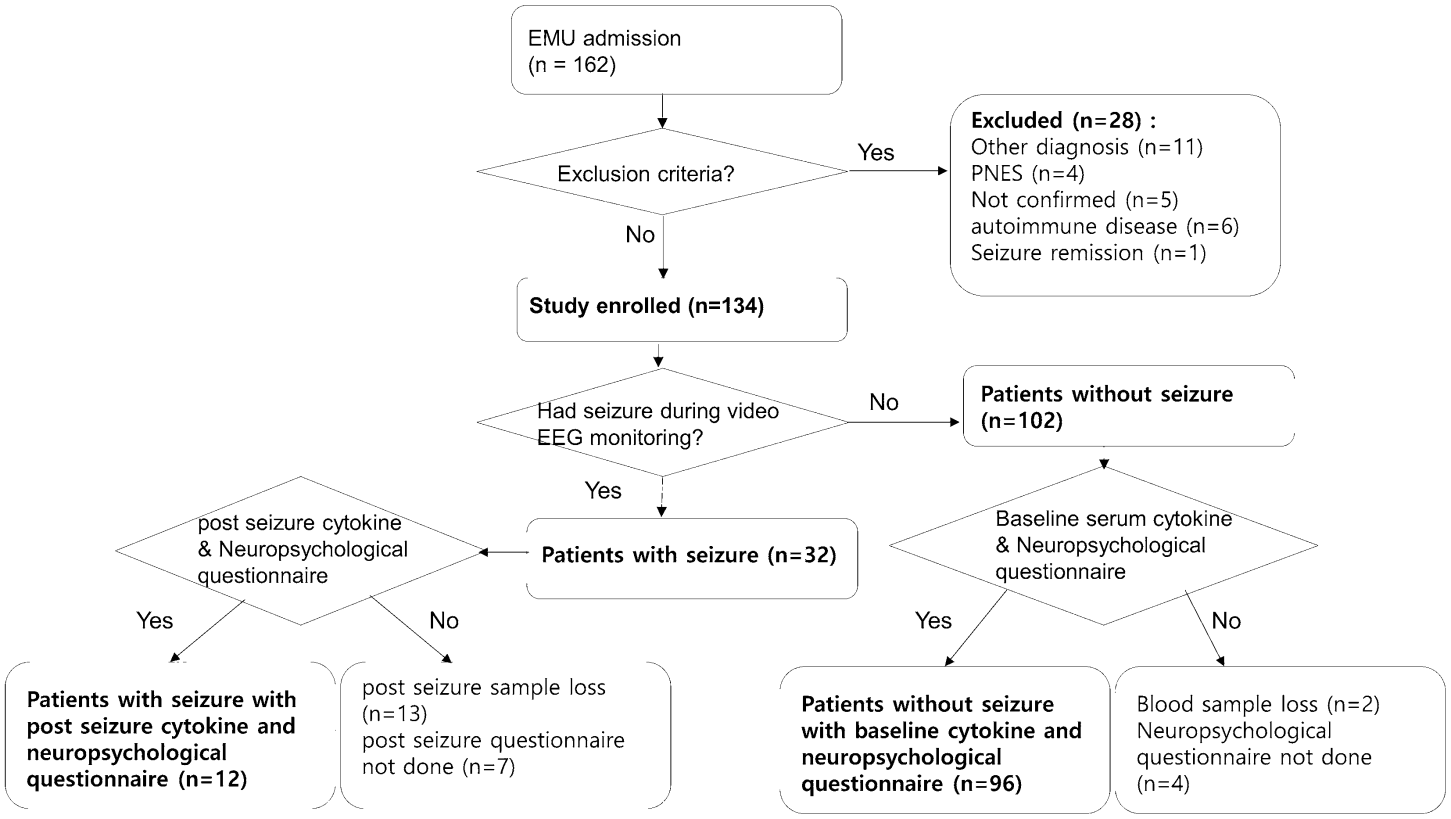
The selection of study subjects among the epilepsy patients. From epilepsy patients who were admitted for video EEG monitoring between July 2019 and December 2020 (n = 162), 134 patients were enrolled. Among them, 32 patients (32.9%) had seizure during the video EEG monitoring and 12 patients had post-seizure cytokine samples and neuropsychiatric questionnaires. Among patients who did not have seizure during video EEG monitoring (n = 102), 96 patients had neuropsychiatric questionnaires and serum cytokine tests.

One hundred and thirty-four patients with epilepsy were enrolled (Table 1, Fig. 1). The median age was 31.0 years (23.0–48.8 years) and 59 (44.0%) were female. All participants had video EEG monitoring for at least 24 h. Thirty-two patients (32.9%) had seizures during the video EEG monitoring, and 11 (8.2%) of them had generalized tonic–clonic seizures (GTCS). The seizure frequency was 1.0/month (0.2–3.5/month), and the period from the last seizure was 22.5 days (6.0–58.5 days).

**Table 1:** Clinical characteristics of study patients.

Subject characteristics	Total (n = 134)
Age—yr	31.0 (23.0–48.8)
Female sex—no. (%)	59 (44.0)
Seizure during video-EEG monitoring—no. (%)	32 (23.9)
Seizure frequency (/month)	1.0 (0.2–3.5)
Period from the last seizure (days)	22.5 (6.0–58.5)
**Epilepsy syndrome type—no. (%)**	
TLE	74 (55.2)
FLE	12 (9.0)
PLE	8 (6.0)
OLE	3 (2.2)
GE	27 (20.1)
Multifocal/unknown	10 (7.5)
**Epilepsy etiology—no. (%)**	
Idiopathic	91 (67.9)
Structural	39 (29.1)
Genetic	1 (0.7)
Post infectious	3 (2.2)
Disease onset—yr	20 (14.0–40.5)
Disease duration—yr	6 (1–15)
Number of AEDs	1 (1.0–2.0)
**Baseline cytokine (n = 132)**	
IL-1β (pg/mL)	4.1 (3.4–4.6)
IL-2 (pg/mL)	34.6 (14.4–248.5)
IL-6 (pg/mL)	6.2 (5.0–8.5)
IFN-γ (pg/mL)	18.5 (14.8–23.8)
CCL2 (pg/mL)	88.9 (72.9–118.0)
CCL5 (pg/mL)	323.7 (198.5–447.3)

TLE: temporal lobe epilepsy; FLE: frontal lobe epilepsy; PLE: parietal lobe epilepsy; OLE: occipital lobe epilepsy; GE: generalized epilepsy; AED: antiepileptic drugs; IL: interleukin; IFN-γ: interferon-gamma; CCL: chemokine motif ligand.

Data are reported as the number (percentage), or as the median (interquartile range, IQR).

The epilepsy syndromes included temporal lobe epilepsy (74, 55.2%), generalized epilepsy (27, 20.1%), frontal lobe epilepsy (12, 9.0%), multifocal/unknown epilepsy (10, 7.5%), parietal lobe epilepsy (8, 6.0%), and occipital lobe epilepsy (3, 2.2%). The most common etiology was idiopathic (91, 67.9%), followed by structural (39, 29.1%), post-infectious (3, 2.2%) and genetic (1, 0.7%). The median age of disease onset was 20 years (14.0–40.5 years), and the median period of disease was 6.0 years (1.0–15.0 years). The median number of antiepileptic drugs (AED)s taken by patients at the time of the video EEG monitoring was 1.0 (1.0–2.0).

We measured the baseline cytokine levels of 132 patients, and the median level was interleukin-1β (IL-1β) 4.1 pg/mL (3.4–4.6 pg/mL), interleukin-2 (IL-2) 34.6 pg/mL (14.4–248.45 pg/mL), interleukin-6 (IL-6) 6.2 pg/mL (5.0–8.5 pg/mL), Interferon-gamma (IFN-γ) 18.5 pg/mL (14.8–23.8 pg/mL), chemokine ligand (CCL) 2 88.9 pg/mL (72.9–118.0 pg/mL), and CCL5 323.7 pg/mL (198.5–447.3 pg/mL).

Comparing patients who had seizures with those who did not have seizures during the video-electroencephalography (EEG) monitoring (Supplementary Table 1), it was found that structural epilepsy was more frequent (*p*-value = 0.002), disease duration was longer (*p*-value < 0.001), and the number of AEDs were higher (*p*-value < 0.001) in the seizure group. However, there were no significant differences relating to age (*p*-value = 0.230), sex (*p*-value = 0.110), seizure frequency (*p*-value = 0.097), and proportion of generalized epilepsy (*p*-value = 0.179).

### Neuropsychiatric symptoms of epilepsy patients

We received the neuropsychiatric questionnaire responses (Hospital Anxiety and Depression Scale (HADS)^19^, Neuropsychiatric Inventory–Questionnaire (NPI-Q)^20^, and Quality of Life in Epilepsy (QOLIE)-31^21^) of 129 patients at admission (Table 2). On the HADS questionnaire, patients with anxiety (HADS-A ≥ 8) were 19 (14.7%), and depression (HADS-D ≥ 8) were 18 (14.0%). The median HADS-A score was 2.0 (0.0–6.0) and the HADS-D score was 3.0 (0.0–6.0). The median NPI-Q severity was 1.0 (0.0–4.0), and distress (n = 82) was 1.0 (0.0–4.0). Depression/dysphonia was the most common (42, 32.6%) of the psychiatric symptoms, followed by irritability/lability (35, 27.1%), sleep/night-time behavior (29, 22.5%) and apathy/indifference (25, 19.4%). The QOLIE-31 was administered to 80 patients, and the median score was 32.5 (25.9–42.2).

**Table 2:** Baseline neuropsychiatric symptoms in patients.

Subject characteristics	Total (n = 129)
HADS-A ≥ 8—no. (%)	19 (14.7)
HADS-D ≥ 8—no. (%)	18 (14.0)
NPI-Q severity	1.0 (0–4.0)
NPI-Q distress (n = 85)	1.0 (0–4.0)
**NPI-Q—no. (%)**	
Delusion	10 (7.8)
Hallucination	8 (6.2)
Agitation/aggression	17 (13.2)
Depression/dysphonia	42 (32.6)
Anxiety	13 (10.1)
Elation/euphoria	7 (5.4)
Apathy/indifference	25 (19.4)
Disinhibition	18 (14.0)
Irritability/lability	35 (27.1)
Aberrant motor behavior	9 (7.0)
Sleep/night-time behavior	29 (22.5)
Appetite/eating disorder	19 (14.7)
**QOLIE-31 (n = 80)**	32.5 (25.9–42.2)
Seizure worry	32.3 (20.0–52.7)
Overall QOL	27.5 (16.0–28.5)
Emotion	36.0 (12.0–49.0)
Energy	55.0 (38.8–70.0)
Cognition	21.4 (16.7–35.3)
Medication	66.7 (58.3–77.7)
Social function	40.0 (35.8–40.0)

HADS-A: Hospital Anxiety and Depression Scale-Anxiety; HADS-D: Hospital Anxiety and Depression Scale-Depression; NPI-Q: Neuropsychiatric Inventory-Questionnaire; QOL: Quality of life.

Data are reported as the number (percentage), or as the median (interquartile range, IQR).

Twelve patients from the seizure group answered the post-seizure neuropsychiatric questionnaire. On the HADS questionnaire, the number of patients with anxiety (HADS-A ≥ 8) was 1 (8.3%), and depression (HADS-D ≥ 8) was 1 (8.3%). The median of NPI-Q severity was 0.

### Cytokine and seizures in epilepsy patients

**Figure 2:**
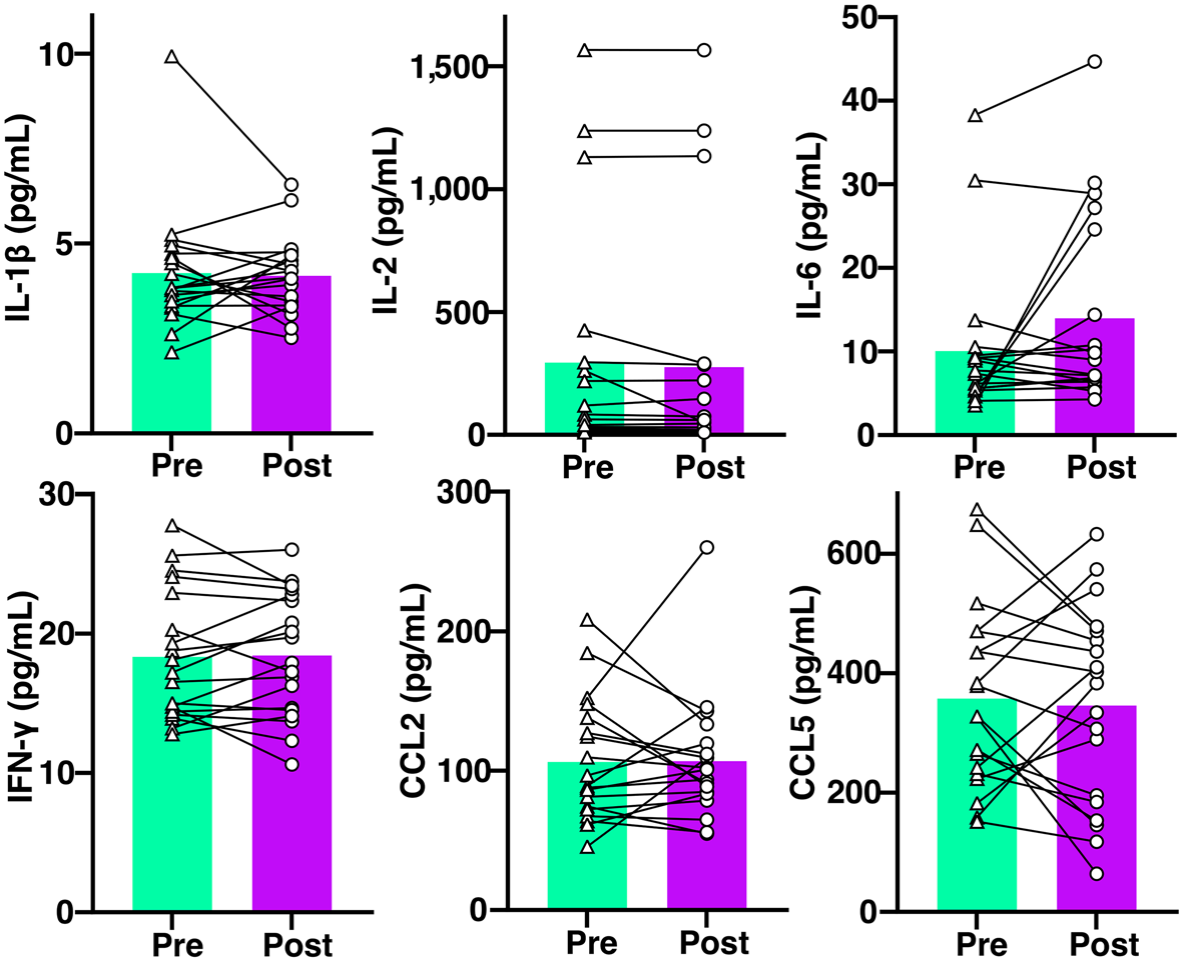
Difference of cytokine levels between baseline and after a seizure. Among the patients who had a seizure during the video EEG monitoring, the change of the cytokine levels between baseline and post-seizure cytokine was analyzed. The IL-1β, IL-2, IL-6, IFN-γ, CCL2, and CCL5 were all not significantly changed before the seizure.

To evaluate the relationship between cytokines and seizures in epilepsy patients, we compared the baseline cytokine levels between patients who had seizures with those who did not during the video EEG monitoring (Supplementary Table 1). There was no significant difference between the baseline levels of all the cytokines. We compared post-seizure cytokines of 19 patients with those of their baseline levels (Fig. 2), and the difference between the cytokine levels of the baseline and post-seizure cytokine levels was not significant. Among 11 patients who had GTCS during admission, we measured the post-seizure cytokines of 7 patients, and there was also no significant change after a seizure. As the pattern of alteration seemed to be diverse in each participant, further analysis was carried out across the clinical characteristics of patients. This revealed that in the group in which IL-2 and IL-6 increased more than 10% from the baseline after seizures, all patients had GTCS, and the proportion of GTCS was higher (*p*-value = 0.013 and 0.005, retrospectively). (Supplementary Table 2).

**Figure 3:**
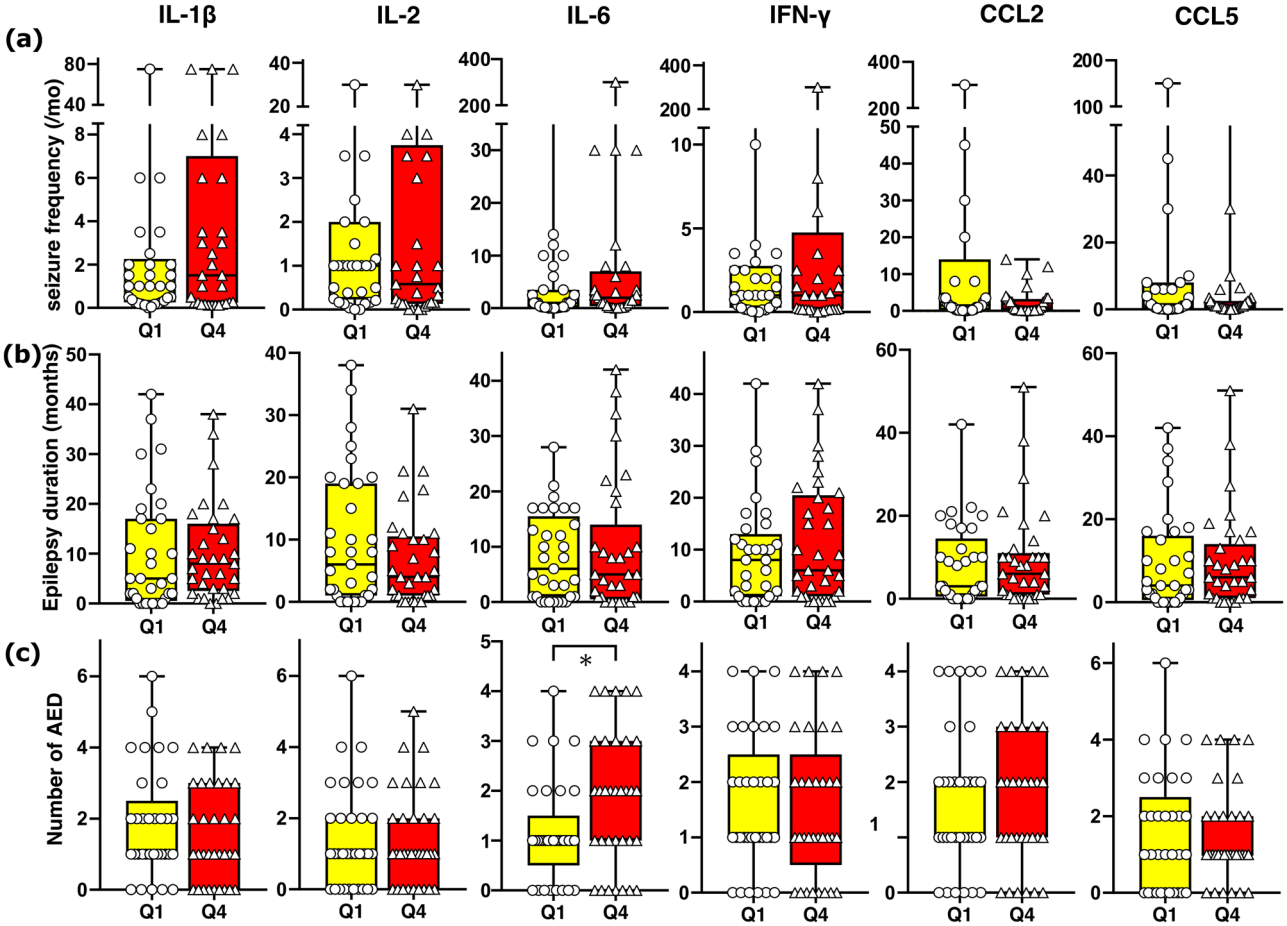
Seizure frequency, disease duration, and the number of AEDs and cytokines. We compared seizure frequency, disease duration, and the number of AEDs of lower (Q1) and upper quartile (Q4) of IL-1β, IL-2, IL-6, IFN-γ, CCL2, and CCL5. There was no significant difference in seizure frequency between Q1 and Q4 of IL-1β, IL-2, IL-6, IFN-γ, and CCL5, except on CCL2, seizure frequency was lower in Q4 (*p*-value = 0.039). The disease duration was longer in Q4 of IL-6 (*p*-value = 0.018). There was no significant difference of AED numbers between Q1 and Q4.

In addition, we compared seizure frequency with disease duration, and the number of AEDs in the upper (Q4) and lower (Q1) quartile of each cytokine (Fig. 3). There was no significant difference in seizure frequency between Q1 and Q4 of IL-1β, IL-2, IL-6, IFN-γ, and CCL5. Seizure frequency was, however, lower in Q4 of CCL2 (Q1 1.5/month (0.2–8.0) and Q4 0.6/month (0.2–3.0), *p*-value = 0.039). The disease duration was not significantly different between Q1 and Q4 of all cytokines. The number of AEDs was higher in Q4 of IL-6 (Q1 1.0 years (1.0–1.0) and Q4 2.0 years (1.0–3.0), *p*-value = 0.027).

### Neuropsychiatric symptoms and cytokines

**Figure 4:**
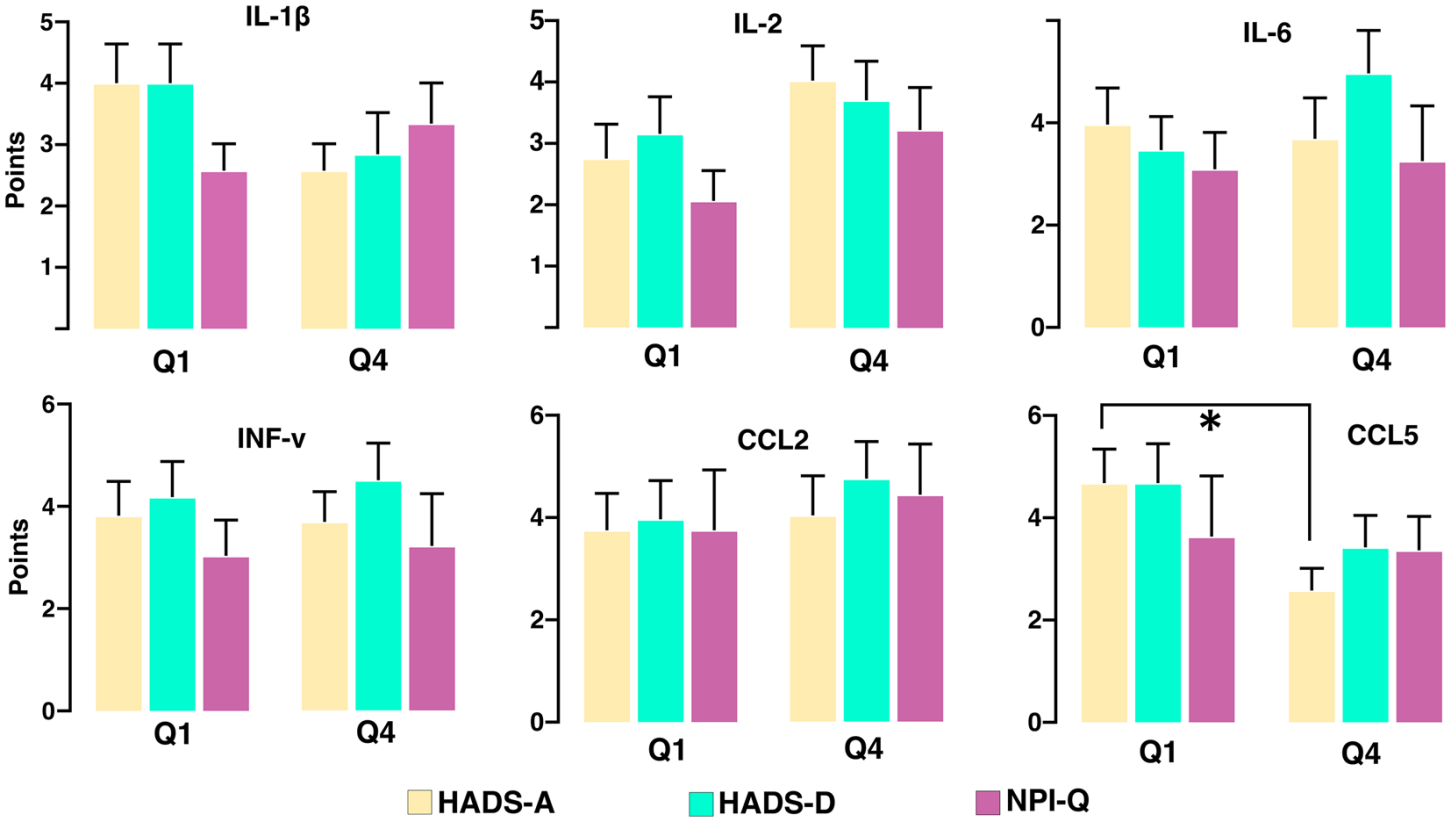
Difference of neuropsychological symptoms between upper and lower quartile of cytokines. We compared the baseline HADS-A, HADS-D, and NPI-Q severity score between lower (Q1) and upper quartile (Q4) of IL-1β, IL-2, IL-6, IFN-γ, CCL2, and CCL5. HADS-A was lower in Q4 of CCL5 (*p*-value = 0.021), and there was no significant difference of HADS-A and HADS-D score between Q1 and Q4 of IL-1β, IL-2, IL-6, IFN-γ, and CCL2. The sum of the NPI-Q severity was different between Q1 and Q4 of all cytokines* is for *p*-value < 0.05.

To evaluate whether neuropsychiatric symptoms are related to cytokine levels, we compared the baseline HADS-A, HADS-D, NPI-Q, and QOLIE-31 scores between Q1 and Q4 of each cytokine (Fig. 4). The patients with anxiety (HADS-A ≥ 8) were less common in Q4 of CCL5 (*p*-value = 0.042). The score of HADS-A was also lower in Q4 of CCL5 (Q1 4.5 (1.0–7.0) and Q4 2.0 (0–4.0), *p*-value = 0.016). There was, however, no significant difference between HADS-A and HADS-D scores between Q1 and Q4 of IL-1β, IL-2, IL-6, IFN-γ, and CCL2. Also, there was no significant difference in the proportion of patients with anxiety (HADS-A ≥ 8) and depression (HADS-D ≥ 8) between Q1 and Q4 of IL-1β, IL-2, IL-6, IFN-γ, and CCL2. The median of the NPI-Q severity and distress score did not show significant differences between Q1 and Q4 of all cytokines. However, on each question of the NPI-Q (Supplementary Table 3), the severity of depression/dysphonia reflected in question 4 was higher in Q4 of CCL2 (Q1 0 (0–1.0) and Q4 1.0 (0–2.3); *p*-value = 0.024). The QOLIE-31 was not different between Q1 and Q4 of all cytokines.

## Discussion

In this study, psychiatric symptom evaluations demonstrated that the Q4 of CCL5 had lower HADS-A scores and anxiety was less common in the Q4 of CCL5. Additionally, the Q4 of CCL2 revealed a higher severity of depression on the NPI-Q. This finding indicates that inflammatory processes including CCL2 and CCL5 may be related to psychiatric symptoms in epilepsy patients.

There are increasing evidence that inflammatory mechanisms contribute to initiating or exacerbating psychiatric disorders^16–18^. Previous studies with major depressive disorder, schizophrenia, and generalized anxiety disorder showed that higher levels of IL-1β, IL-2, IL-6, and CCL2 were related to the severity of psychiatric symptoms^16–18,22,23^. However, there are only a few studies that suggested the relationship between psychiatric symptoms in epilepsy patients and inflammation. Several studies on epilepsy revealed that IL-1β is related to the pathomechanism of epilepsy-related depression^12–15^. Clinical and animal studies of an epilepsy model, where anti-inflammatory drugs such as celecoxib (cyclooxygenase inhibitor), Anakinra (IL-1β antagonist), and minocycline (microglial inhibitor) ameliorated depression, also support this hypothesis^14,24–26^. The result of one human study showed a higher blood level of IL-1β in epilepsy with depression compared with that without depression^15^.

While this study did not demonstrate a relationship between cytokines with anxiety, depression, and the overall severity of psychiatric symptoms as reflected by responses to the NPI-Q, it did suggest a potential relationship between inflammation and psychiatric symptoms in epilepsy patients. Additionally, seizures or epilepsy were all confirmed by video EEG monitoring, and detailed psychiatric symptoms were evaluated by means of responses to neuropsychiatric questionnaires. Considering inflammatory processes also have an important role in epileptogenesis, inflammation may serve as a connector between epilepsy and psychiatric disorders^12–15^.

CCL2 is a proinflammatory chemokine which causes neuroinflammation by promoting the chemotaxis of peripheral macrophages, T cells, and dendritic cells to the brain^22,23^. It is a possible mediator of the pathogenesis of depression since previous studies have demonstrated that serum CCL2 is elevated in major depressive disorder and reduced after antidepressant treatment^23,27^. This finding elucidates how elevated CCL2 may be related to depression.

CCL5 is also a proinflammatory chemokine which attracts T cells and eosinophils into inflammatory sites^28,29^. Though several reports have shown that anxiety is associated with high levels of pro-inflammatory cytokines^18,22,30^, there are reports that some chemokines, especially CCL27 and CCL11, negatively correlate with anxiety^31,32^. Since chronic stress may induce suppression of the immune response, it may indicate a chronic stress-induced compromised state^28,31,32^. This mechanism also suggests why the lower quartile of CCL2 showed a higher seizure frequency. Recurrent seizures may result in chronic stress-induced immune suppression.

Since neuroinflammation is related to psychiatric comorbidities in epilepsy patients, serum cytokine could be a biomarker to be used as a screening test or to evaluate a treatment effect. Previous studies have shown that the cytokine level is reduced after medical treatment, and related to disease severity in patients with psychiatric disorders^17,25,27^. Additionally, several studies have suggested that anti-inflammatory treatments could improve psychiatric symptoms, especially depression^22,24–26^. Psychiatric comorbidities can worsen medical and surgical outcomes and reduce tolerance of AEDs in epilepsy patients^33^. After further research into anti-inflammatory treatments for psychiatric comorbidities in epilepsy patients, it may be employed to treat severe psychiatric symptoms in epilepsy.

However, we did not find a statistically significant association between the acute alteration of serum cytokines and clinical information relating to seizure activity in epilepsy patients. We only found a relationship between IL-6 and the number of AEDs. Cytokine levels did not change after seizures, but increased IL-2 and IL-6 after seizures were related to a higher rate of GTCS. The cytokine levels may have varied over time after the seizures, thus producing a similar result to those of previous studies which revealed only increased IL-2 and IL-6 levels after seizures^4–6^.

Our study has several limitations. First, this study did not show cytokine changes after seizures, despite previous studies suggesting pro-inflammatory cytokine increases after seizures^4–6^. The study garnered 32 patients who were observed to have seizures during admission. Hence there was a limitation in analyzing the change of cytokines after seizures due to the small sample size. In addition, the timing of post-seizure sampling was not standardized within a boundary of routine clinical practice and it was done grossly within 6 h after a seizure. Also, cytokines are known to vary with sample timing, storage, and handling. The sample timing and difficulty of cytokine measurements might limit the analysis^34^. However, since the patients had frequent seizure, the baseline cytokine could be increased due to recurrent seizure. The post-seizure cytokine might not have changed due to this chronic upregulation. Further study with standardized post-seizure sampling time in the patients with relatively low seizure frequency is needed. Second, we did not demonstrate the relationship of HADS-A, HADS-D, and total NPI-Q severity score with cytokines. However, since each question of the NPI-Q has different clinical implications, the relationship between NPI-Q sub-items (depression) severity and cytokine levels revealed a relationship between elevated cytokine levels with each psychiatric symptom. Additionally, this study excluded patients who were taking psychiatric drugs in order to rule out the potential effect of medications on cytokines. Thus, patients with more severe psychiatric symptoms were not included in the analysis, because patients who were diagnosed with psychiatric disease or taking psychiatric drugs are more likely to have severe symptoms. This point might cause an underevaluation of the relationship between cytokines and psychiatric symptoms in the study. Further, AED was not controlled among the study patients. Various AEDs have an impact on cytokine and psychiatric symptoms^35,36^, so the effect of AEDs on both cytokine and psychiatric symptoms cannot be excluded. Prospective well-designed research with a homogenous AED population should be carried out. Last, we only included patients who were admitted for video-EEG monitoring. As video-EEG monitoring is mostly done on patients with higher seizure severity, patients with mild symptoms might be excluded. Further study including outpatients, could evaluate whether psychiatric symptoms in epilepsy patients with milder seizure severity are related to cytokine.

In conclusion, this study showed cytokines’ levels are related to psychiatric symptoms in epilepsy patients. We investigated multiple serum cytokines and multiple psychiatric phenotypes in epilepsy patients and their relationship. The results suggest inflammatory processes may be related to psychiatric symptoms in epilepsy patients and serve as a mediator between epilepsy and psychiatric symptoms. More research involving longitudinal large cohorts of epilepsy patients for validation is warranted to evaluate neuroinflammation as common pathogenesis of epilepsy and psychiatric disorders. Understanding the role of neuroinflammation in epilepsy, beyond seizure or epilepsy, would help to prioritize the target of an anti-inflammatory strategy.

## Methods

### Ethics declarations

This study was approved by the Seoul National University Hospital Institutional Review Board (1906-107-1041) and followed the principles of the Declaration of Helsinki. We obtained written informed consent from all participants, and the information of patients was sufficiently anonymized. We confirm that we have read the Journal's position on issues involved in ethical publication and affirm that this report is consistent with those guidelines.

### Patient enrollment

We screened epilepsy patients who were admitted for video-EEG monitoring at Seoul National University Hospital between July 2019 and December 2020. All patients who were diagnosed as epileptic, both clinically and by EEG, were included in the study. Patients with any autoimmune disease including autoimmune encephalitis, and an active infection which might affect the cytokine levels, were excluded. Patients with a history of a psychiatric disorder or mental retardation and those who were on psychiatric medication (antidepressant, anxiolytics, and antipsychotics) were excluded due to the effect of these variables on the neuropsychiatric evaluation.

We reviewed demographic data, disease onset, disease duration, seizure frequency (/month), number of AEDs, brain MRI, and video-EEG results. All seizures during admission were confirmed by video-EEG recordings by an experienced neurologist (K.C., K.Y.J., S.K.L., and K.I.P).

### Serum cytokine measurements

We took serum samples of cytokines (IL-1β, IL-2, IL-6, IFN-γ, CCL2, and CCL5) at admission with informed consent. The samples were stored at − 80 °C, and quantification of all the cytokines was based on an enzyme-linked immunosorbent assay (ELISA) using commercially available kits according to the manufacturer’s instructions (BioLegend, San Diego, CA, USA).

Briefly, we prepared a standard solution with Assay Buffer D (BioLegend, San Diego, CA, USA) to make a standard stock solution. The reconstituted standard was left at room temperature for 15–20 min and mixed completely. We prepared the 500 μL of the top standard by adding the standard stock solution to Assay Buffer D and performed serial dilution. After that, we washed the plate 4 times using a wash buffer and added 50 μL of matrix E to standard wells and 50 μL of Assay Buffer D to sample wells. After adding 50 μL of diluted standards to standard wells and 50 μL of samples to sample wells, these were incubated for 2 h before being shaken. The concentration of cytokines was calculated based on the standard curves provided with the kits, and the results were expressed in pg/ml. For ELISA, all samples were tested in duplicate and average values were used in the analysis.

Patients who had clinical seizures during the video-EEG monitoring repeated laboratory tests for all cytokines within 6 h after their seizures.

### Neuropsychiatric evaluation

We received the neuropsychiatric questionnaires, Hospital Anxiety and Depression Scale (HADS)^19^, Neuropsychiatric Inventory–Questionnaire (NPI-Q)^20^, and Quality of Life in Epilepsy (QOLIE)-31^21^ at admission. A HADS-Anxiety (HADS-A) score ≥ 8 and a HADS-Depression (HADS-D) score ≥ 8 were considered to be indications of anxiety and depression respectively^19^.

When a patient had a seizure during admission, we received the post-seizure neuropsychiatric questionnaire (HADS and NPI-Q) 1–4 weeks after the seizure, when they visited our neurology clinic, or by telephone.

### Statistical analysis

The results are presented as median (range) or number (%). Independent t-tests were performed to compare patients’ characteristics and baseline cytokine levels between the seizure and non-seizure groups. This included their clinical features, neuropsychiatric questionnaire scores, differences between the Q4 and Q1 of cytokines, and clinical characteristics associated with increased cytokines after seizures. The chi-square test was performed to compare the sex distribution between the seizure and non-seizure groups, and to analyze the clinical characteristics correlating with increased cytokines after seizures. The paired t-test was performed to evaluate the change of cytokine levels before and after seizures. SPSS 25 was used for all statistical analyses, and two-tailed *p*-value < 0.05 were considered statistically significant.

## Supplementary Information

Supplementary Tables.

## Data Availability

The datasets used and analyzed during the current study are available from the corresponding author on reasonable request. All data generated or analyzed during this study are included in this published article and supplementary data. All authors share the raw data provided in supplementary data.
