# Supplementary material for: Neuropsychiatric symptoms and seizure related with serum cytokine in epilepsy patients
Source: Sci Rep. 2022 May 3;12:7138. doi: 10.1038/s41598-022-10865-x (PMC9065058; doi:10.1038/s41598-022-10865-x)
Supplement: Supplementary file 1 — Supplementary Tables. [file 41598_2022_10865_MOESM1_ESM.docx]

**Supplementary Table 1. Patient characteristics and baseline cytokine levels of patients who had seizure or not during video EEG monitoring.**

|  | **Seizure (n=32)** | **No seizure (n=102; cytokine level, n=100)** | ***p*-value** |
| --- | --- | --- | --- |
| Age – yr (range) | 31.0 (25.5–38.8) | 31.0 (21.3–53.0) | 0.230 |
| Female sex – no. (%) | 18 (56.3) | 41 (40.2) | 0.110 |
| Seizure frequency (/month) | 3.5 (1.4–10.0) | 0.5 (0.1–1.9) | 0.097 |
| Generalized epilepsy (%) | 3 (9.4) | 24 (23.5) | 0.179 |
| Structural epilepsy (%) | 17 (53.1) | 24 (23.5) | 0.002 |
| Disease duration – yr | 11.0 (6.0–21.3) | 4.0 (1.0–10.0) | <0.001* |
| Number of AEDs | 2.0 (2.0–3.0) | 1.0 (1.0–2.0) | <0.001* |
| IL-1β (pg/mL) | 4.0 (3.4–4.6) | 4.1 (3.4–4.6) | 0.795 |
| IL-2 (pg/mL) | 45.9 (15.3–328.0) | 34.1 (14.3–221.8) | 0.133 |
| IL-6 (pg/mL) | 7.5 (5.3–9.3) | 6.1 (4.8–8.1) | 0.252 |
| IFN-γ (pg/mL) | 18.6 (14.8–23.2) | 18.5 (14.9–23.9) | 0.217 |
| CCL2 (pg/mL) | 92.9 (78.8–127.6) | 87.6 (71.2–113.1) | 0.349 |
| CCL5 (pg/mL) | 324.3 (222.3–444.7) | 319.7 (185.6–447.3) | 0.466 |

* Data are reported as the number (percentage), or as the median (interquartile range, IQR). AED: antiepileptic drugs; IL: interleukin; IFN-γ: interferon gamma; CCL: chemokine motif ligand. * is for *p*-value < 0.05.

**Supplementary Table 2. Characteristics of patients with increased cytokines after seizure**

|  | **Number of patients (n=19)** | **GTCS** | **Structural epilepsy** | **Disease duration** | **Seizure frequency (/mo.)** | **Number**  **of AEDs** |
| --- | --- | --- | --- | --- | --- | --- |
| **IL-1β** | 7 (36.8) | 1 | 3 | 13.5  (9.0–19.0) | 3.5  (3.0–3.8) | 2.0  (1.5–3.0) |
| *p*-value |  | 0.12 | 0.764 | 0.741 | 0.583 | 0.686 |
| **IL-2** | 3 (15.8) | 3 (100) | 3 (100) | 6.0  (5.5–11.5) | 0.2  (0.2–0.4) | 2.0  (1.5–2.5) |
| *p*-value |  | 0.013* | 0.047 | 0.413 | 0.203 | 0.474 |
| **IL-6** | 9 (47.3) | 9 (100) | 4 | 18.0  (17.0–20.0) | 3.5  (3.5–10.0) | 3.0  (2.0–4.0) |
| *p*-value |  | 0.005* | 0.809 | 0.362 | 0.894 | 0.020* |
| **IFN-γ** | 6 (31.6) | 2 (33.3) | 3 | 14.0  (10.3–20.0) | 3.5  (1.3–3.9) | 2.0  (2.0–2.8) |
| *p*-value |  | 0.829 | 0.876 | 0.730 | 0.092 | 0.816 |
| **CCL2** | 6 (31.6) | 2 (33.3) | 4 | 14.0  (10.3–20.0) | 2.0  (0.5–3.9) | 2.0  (2.0–2.8) |
| *p*-value |  | 0.829 | 0.252 | 0.708 | 0.048* | 0.816 |
| **CCL5** | 7 (36.8) | 2 | 3 | 11.0  (6.5–19.0) | 8.0  (3.8–22.0) | 2.0  (1.5–3.5) |
| *p*-value |  | 0.568 | 0.764 | 0.475 | 0.110 | 0.982 |

* Data are reported as the number (percentage), or as the median (interquartile range, IQR). GTCS : generalized tonic clonic seizure; AED: antiepileptic drugs; IL-1β: interleukin-1beta; IL-2: interleukin-2; IL-6: interleukin-6; IFN- γ: interferon gamma; CCL2: chemokine motif ligand 2; CCL5: chemokine motif ligand 5. * is for *p*-value < 0.05.

**Supplementary Table 3. Severity of Neuropsychiatric Inventory–Questionnaire (NPI-Q) in upper and lower quartile of cytokines.**

|  | **1**  **Delusions** | **2**  **Hallucination** | **3**  **Agitation** | **4**  **Depression** | **5**  **Anxiety** | **6**  **Euphoria** | **7**  **Apathy** | **8**  **Disinhibition** | **9**  **Irritability** | **10**  **Aberrant motor behavior** | **11**  **Nighttime disturbance** | **12**  **Appetite disturbance** |
| --- | --- | --- | --- | --- | --- | --- | --- | --- | --- | --- | --- | --- |
| IL-1β Q1 | 0 (0.0–0.0) | 0 (0.0–0.0) | 0 (0.0–0.0) | 0 (0.0–1.0) | 0 (0.0–0.0) | 0 (0.0–0.0) | 0 (0.0–0.0) | 0 (0.0–0.0) | 0 (0.0–0.0) | 0 (0.0–0.0) | 0 (0.0–1.0) | 0 (0.0–0.0) |
| IL-1β Q4 | 0 (0.0–0.0) | 0 (0.0–0.0) | 0 (0.0–0.0) | 0 (0.0–1.0) | 0 (0.0–0.0) | 0 (0.0–0.0) | 0 (0.0–0.0) | 0 (0.0–0.0) | 0 (0.0–1.0) | 0 (0.0–0.0) | 0 (0.0–0.0) | 0 (0.0–0.0) |
| *p*-value | 0.613 | 0.325 | 0.208 | 0.267 | 0.325 | 0.758 | 0.956 | 0.276 | 0.397 | 0.330 | 0.721 | 0.917 |
| IL-2 Q1 | 0 (0.0–0.0) | 0 (0.0–0.0) | 0 (0.0–0.0) | 0 (0.0–1.0) | 0 (0.0–0.0) | 0 (0.0–0.0) | 0 (0.0–0.0) | 0 (0.0–0.0) | 0 (0.0–0.0) | 0 (0.0–0.0) | 0 (0.0–1.0) | 0 (0.0–0.0) |
| IL-2 Q4 | 0 (0.0–0.0) | 0 (0.0–0.0) | 0 (0.0–0.0) | 0 (0.0–1.0) | 0 (0.0–0.0) | 0 (0.0–0.0) | 0 (0.0–0.0) | 0 (0.0–0.0) | 0 (0.0–1.0) | 0 (0.0–0.0) | 0 (0.0–0.0) | 0 (0.0–0.0) |
| *p*-value | 0.316 | 0.785 | 0.200 | 0.142 | 0.159 | 0.562 | 0.393 | 0.086 | 0.062 | 0.427 | 0.403 | 0.966 |
| IL-6 Q1 | 0 (0.0–0.0) | 0 (0.0–0.0) | 0 (0.0–0.0) | 0 (0.0–1.0) | 0 (0.0–0.0) | 0 (0.0–0.0) | 0 (0.0–0.0) | 0 (0.0–0.0) | 0 (0.0–1.0) | 0 (0.0–0.0) | 0 (0.0–0.3) | 0 (0.0–0.0) |
| IL-6 Q4 | 0 (0.0–0.0) | 0 (0.0–0.0) | 0 (0.0–0.0) | 0 (0.0–1.0) | 0 (0.0–0.0) | 0 (0.0–0.0) | 0 (0.0–0.0) | 0 (0.0–0.0) | 0 (0.0–0.0) | 0 (0.0–0.0) | 0 (0.0–0.0) | 0 (0.0–0.0) |
| *p*-value | 0.340 | 0.103 | 0.854 | 0.704 | 0.825 | 0.658 | 0.612 | 1.0 | 0.878 | 0.137 | 0.930 | 0.152 |

| IFN-γ Q1 | 0 (0.0–0.0) | 0 (0.0–0.0) | 0 (0.0–0.0) | 0 (0.0–0.0) | 0 (0.0–0.0) | 0 (0.0–0.0) | 0 (0.0–1.0) | 0 (0.0–0.0) | 0 (0.0–0.0) | 0 (0.0–0.0) | 0 (0.0–0.0) | 0 (0.0–0.0) |
| --- | --- | --- | --- | --- | --- | --- | --- | --- | --- | --- | --- | --- |
| IFN-γ Q4 | 0 (0.0–0.0) | 0 (0.0–0.0) | 0 (0.0–0.0) | 0 (0.0–1.0) | 0 (0.0–0.0) | 0 (0.0–0.0) | 0 (0.0–0.0) | 0 (0.0–0.0) | 0 (0.0–1.0) | 0 (0.0–0.0) | 0 (0.0–0.0) | 0 (0.0–0.0) |
| *p*-value | 0.155 | 0.973 | 0.824 | 0.413 | 0.399 | 0.057 | 0.438 | 0.775 | 0.950 | 0.451 | 0.096 | 0.876 |
| CCL2 Q1 | 0 (0.0–0.0) | 0 (0.0–0.0) | 0 (0.0–0.0) | 0 (0.0–1.0) | 0 (0.0–0.0) | 0 (0.0–0.0) | 0 (0.0–0.0) | 0 (0.0–0.0) | 0 (0.0–0.0) | 0 (0.0–0.0) | 0 (0.0–1.0) | 0 (0.0–0.0) |
| CCL2 Q4 | 0 (0.0–0.0) | 0 (0.0–0.0) | 0 (0.0–0.0) | 1.0 (0.0–2.3) | 0 (0.0–0.0) | 0 (0.0–0.0) | 0 (0.0–0.0) | 0 (0.0–0.3) | 0 (0.0–1.3) | 0 (0.0–0.0) | 0 (0.0–0.0) | 0 (0.0–0.0) |
| *p*-value | 0.195 | 0.194 | 0.438 | 0.024* | 0.262 | 0.779 | 0.409 | 0.194 | 0.132 | 0.845 | 0.223 | 1.0 |
| CCL5 Q1 | 0 (0.0–0.0) | 0 (0.0–0.0) | 0 (0.0–0.0) | 0 (0.0–0.8) | 0 (0.0–0.0) | 0 (0.0–0.0) | 0 (0.0–0.0) | 0 (0.0–0.0) | 0 (0.0–0.8) | 0 (0.0–0.0) | 0 (0.0–0.8) | 0 (0.0–0.0) |
| CCL5 Q4 | 0 (0.0–0.0) | 0 (0.0–0.0) | 0 (0.0–0.0) | 0 (0.0–2.0) | 0 (0.0–0.0) | 0 (0.0–0.0) | 0 (0.0–1.0) | 0 (0.0–0.0) | 0 (0.0–0.0) | 0 (0.0–0.0) | 0 (0.0–1.0) | 0 (0.0–0.0) |
| *p*-value | 0.744 | 0.490 | 0.410 | 0.436 | 0.782 | 0.618 | 0.745 | 0.574 | 0.369 | 0.927 | 0.734 | 0.874 |

* Data are reported as the median (interquartile range, IQR). IL-1β: interleukin-1beta; IL-2: interleukin-2; IL-6: interleukin-6; IFN- γ: interferon gamma; CCL2: chemokine motif ligand 2; CCL5: chemokine motif ligand 5. * is for *p*-value < 0.05.
